# Modeling frameworks in nutritional epidemiology matter: comparing isotemporal and time-lagged Bayesian and frequentist approaches of carbohydrate intake and adiposity

**DOI:** 10.3389/fnut.2025.1700898

**Published:** 2025-12-18

**Authors:** Sean Titensor, Joshua L. Ebbert, David Camacho, Karen A. Della Corte, Antonio L. Palmeira, R. James Stubbs, Graham Horgan, Berit L. Heitmann, Dennis Della Corte

**Affiliations:** 1 Department of Physics and Astronomy, Brigham Young University, Provo, UT, United States; 2 Department of Mathematics, Brigham Young University, Provo, UT, United States; 3 Department of Nutrition, Dietetics, and Food Science, Brigham Young University, Provo, UT, United States; 4 CIDEFES, Universidade Lusófona, Lisbon, Portugal; 5 CIFI2D, Universidade do Porto, Lisbon, Portugal; 6 Appetite Control and Energy Balance Group, School of Psychology, University of Leeds, Leeds, United Kingdom; 7 Biomathematics and Statistics Scotland, Aberdeen, United Kingdom; 8 Research Unit for Diet and Health, The Parker Institute, Frederiksberg and Bispebjerg Hospital, Copenhagen, Denmark; 9 Section for General Practice, Department of Public Health, University of Copenhagen, Copenhagen, Denmark; 10 The Boden Group, Charles Perkins Centre, University of Sydney, Sydney, NSW, Australia

**Keywords:** Bayesian inference, frequentist statistics, longitudinal modeling, time-lagged analysis, isotemporal substitution, carbohydrate intake, adiposity, statistical modeling

## Abstract

**Background:**

Understanding how different modeling strategies affect associations in nutritional epidemiology is critical, especially given the temporal complexity of dietary and health data.

**Objective:**

To compare how different modeling frameworks—including isotemporal versus time-lagged designs and frequentist versus Bayesian inference—affect estimated associations between carbohydrate subtypes and adiposity.

**Methods:**

Longitudinal data of 415 adults from the NoHoW Study were used to investigate associations between four carbohydrate predictors (free sugars, intrinsic sugars, starch, and dietary fiber) and three indices of adiposity (body fat percentage, BMI, and waist circumference) as outcomes. Four statistical approaches were used contrasting frequentist and Bayesian methods across both isotemporal (concurrent measurement) and time-lagged (6-month temporal shift) frameworks. To specifically evaluate *change* in adiposity outcomes over time, we implemented additional baseline-adjusted longitudinal models.

**Results:**

Isotemporal and time-lagged models showed directional agreement for nearly all associations; in all but one case, the models either aligned in the direction of the association or differed only in relation to the null. However, time-lagged models identified statistically significant associations and produced larger effect sizes for body fat outcomes and for starch and fiber predictors. Other associations, including intrinsic and free sugars, were weaker and varied with model specification, losing statistical support under time-lagged models. Frequentist models exhibited greater variation across temporal frameworks, including one directional shift among significant associations. Effect estimates were substantially attenuated after adjustment for baseline adiposity.

**Discussion:**

Time-lagged modeling shifted associations between carbohydrate intake and anthropometric outcomes, with increased effect sizes and additional significant associations for starch and fiber, and fewer statistically significant associations for intrinsic and extrinsic sugars. In contrast to frequentist models, Bayesian models yielded more stable and consistent estimates across time-lagged and isotemporal frameworks, showing no differences in the directions of associations across temporal frameworks. Models unadjusted for baseline adiposity overstate dietary impacts; including baseline adiposity is essential to isolate true diet-change effects from initial weight.

**Conclusion:**

Our findings suggest that incorporating temporal structure, especially through Bayesian models, can uncover relevant relationships that concurrent models may overlook. This study demonstrates that model specification, both in temporal framework and statistical approach, meaningfully influences both the detection and interpretations of associations in nutritional epidemiology.

## Introduction

Analyzing associations between dietary exposures and health outcomes in observational studies presents unique methodological challenges (1, 2). In nutritional epidemiology, researchers often contend with temporally misaligned measurements and reverse causality, factors that can complicate the interpretation of findings (3). Given these complexities, analytical decisions, such as how time and exposure are modeled, can substantially influence study findings and the conclusions drawn from them.

One key modeling decision is how to represent the temporal relationship between exposures and outcomes (4). Isotemporal models estimate associations using concurrent measurements, while time-lagged models use prior exposures to predict future outcomes, possibly increasing causal interpretability (5). For example, observed associations between sugar intake and weight status may reflect compensatory dietary changes made in response to weight gain, rather than true associations between sugar intake and adiposity. Time-lagged analyses, in contrast, impose a temporal sequence by modeling exposures at time *t* and outcomes at time *t + 1*, which may more closely approximate the logic of intervention studies, though it comes at the cost of reduced sample size and increased model complexity (6). Although time-lagged frameworks may better align with hypothesized biological pathways, they are less commonly used and rarely compared directly with isotemporal models (7).

A second important modeling decision involves the inferential framework used to estimate associations. Frequentist methods remain the standard in nutritional science, relying on *p-*values, hypothesis tests, and confidence intervals to evaluate evidence (8–10), and offer limited insight into effect probabilities. Bayesian inference, by contrast, provides a more flexible alternative by integrating prior knowledge with observed data to yield posterior distributions, enabling direct probability estimates about effect size and intuitive measures of uncertainty (11–14). Despite these advantages, Bayesian methods are underutilized in nutritional epidemiology, in part due to historical computational barriers (3, 8–10, 15). However, recent advances in probabilistic programming (e.g., Stan, PyMC) have made Bayesian modeling increasingly accessible and scalable for complex data structures (16–20).

While prior studies have examined temporal structure or inferential frameworks independently, few have explored how these modeling choices jointly influence findings (21–23). This is a critical gap, as the intersection of temporal and inferential decisions may substantially affect the strength, direction and interpretation of associations in longitudinal nutrition data. Our study aims to address this by directly comparing isotemporal and time-lagged models under both Bayesian and frequentist approaches.

Additionally, we examine the effect of baseline adjustment for BMI on the results of time-lagged models. If heavier participants at baseline prefer certain carbohydrate subtypes and then adjust those choices differently over time, not controlling for baseline adiposity means the estimated diet effect may simply reflect initial weight-related behaviors rather than true dietary change. Including baseline adiposity as a covariate removes this confounding and isolates the relationship between diet change and adiposity. Although some argue that baseline adjustment can “block” part of an exposure’s effect, that only applies when the exposure causally influences the baseline measure. In our study, diet and adiposity measures are assessed simultaneously at baseline, so adjusting for baseline adiposity controls for pre-existing adiposity differences without removing any causal pathway.

In this study, we perform a cross-framework comparison of isotemporal and time-lagged models using both frequentist and Bayesian approaches. We apply these methods to longitudinal data from the NoHoW Study (24), examining associations between dietary carbohydrate subtypes—free sugars, intrinsic sugars, starch, and fiber—and adiposity-related outcomes (25). Building on our prior work, which established carbohydrate–adiposity associations using a single Bayesian framework and a predefined causal DAG (26), the current study systematically evaluates whether variations in temporal specification and statistical framework materially influence inferred associations, effect magnitudes, or the degree of statistical certainty for identical exposures and outcomes. This work offers a structured assessment of how temporal alignment and inferential approach interact to shape interpretations in nutritional epidemiology.

## Methods

### Study design and participants

This study draws on data from the NoHoW (Navigating to a Healthy Weight) trial, a multicenter, 18-month, randomized, controlled European study designed to evaluate an evidence-based digital toolkit aimed at supporting self-regulation, motivation, and emotion regulation for weight-loss maintenance. Adult participants (≥18 years) were eligible if they had previously lost at least 5% of their body weight and had a pre-weight-loss BMI ≥ 25 kg/m^2^. Recruitment occurred between March 2017 and March 2018 across sites in the UK, Denmark, and Portugal. Further methodological details of the NoHoW trial are available elsewhere (27). Our analysis included 415 participants who provided complete data on dietary intake, anthropometric measures, and covariates across four time points: baseline, 6 months, 12 months, and 18 months (24). The study complies with relevant EC legislation, international conventions, and declarations relating to ethical research practices (28). All participants provided written informed consent.

### Dietary exposure and anthropometric outcome variables

Dietary exposures of interest were four distinct carbohydrate subtypes: free sugars (non-milk extrinsic sugars, or NMES), intrinsic sugars, starch, and fiber. These were assessed within 7 days of all four clinical investigation dates (baseline, 6 months, 12 months, and 18 months) on at least four consecutive days, including one weekend day, using 24-h web-based dietary recalls. The mean values for each participant across all recall days were then energy-adjusted using the residual method to control for total caloric intake. All exposures were standardized as *z*-scores prior to modeling. Outcome variables included three anthropometric markers—body fat percentage, body mass index (BMI), and waist circumference—measured at each time point. Covariates included age, sex, study center, dietary protein, alcohol, saturated fat, fiber, and residual energy intake. Baseline comorbidities such as hypertension and diabetes were considered as potential confounders but not included in alignment with previous work (26).

The self-reported dietary assessments were conducted using the web-based tool INTAKE24 (29), which measures food intake and estimates daily energy and nutrient intake from standardized food tables. Anthropometric variables were measured by trained research staff after a 10–12 h overnight fast (27). Fasting bioimpedance (ImpediMed™ SFB7, Queensland, Australia) was used to estimate body composition, including body fat percentage. Body height and weight (Seca 704s, SECA, Germany) were measured with participants barefoot and in light clothing, respectively, and used to determine participant BMI with the standard equation: kg/m^2^. The waist circumference measurement was taken against bare skin or light clothing at the midpoint between the lower margin of the last palpable rib and the top of the iliac crest at the end of a normal expiration to the nearest 0.1 cm as recommended by the WHO (27, 30).

### Temporal frameworks: isotemporal and time-lagged

We examined the influence of temporal structure on model estimates using two distinct longitudinal modeling frameworks: isotemporal and time-lagged. In the isotemporal framework, dietary exposures and outcomes were measured concurrently at each time point. This approach retained all repeated measures, allowing within-subject variation to be accounted for using participant-specific random effects. In contrast, the time-lagged framework aligned dietary exposures with outcome measures collected 6 months later; due to data restrictions, shorter intervals were not available, which could have provided greater biological predictive power and reduced potential seasonal confounding. Specifically, dietary intake at baseline, 6 months, and 12 months was used to predict outcomes at 6, 12, and 18 months, respectively. This alignment removed the first outcome and last predictor time points to preserve temporal directionality, simulating causal precedence between exposure and outcome (6, 31). Table 1 summarizes the alignment structure for each modeling framework.

**Table 1:** Alignment of isotemporal and time-lagged outcomes and predictors.

Temporal framework	Time 1	Time 2	…	Time *n*
Isotemporal				
Outcomes	✓	✓	✓	✓
Predictors	✓	✓	✓	✓
Time-lagged				
Outcomes	×	✓	✓	✓
Predictors	✓	✓	✓	×

Comparison of data availability for outcomes and predictors over time between isotemporal and time-lagged temporal frameworks. The time-lagged approach has excluded data for outcomes at Time 1 and predictors at final Time *n*, reducing the overall data available for analysis compared to the isotemporal framework.

### Statistical models: frequentist and Bayesian approaches

**Figure 1:**
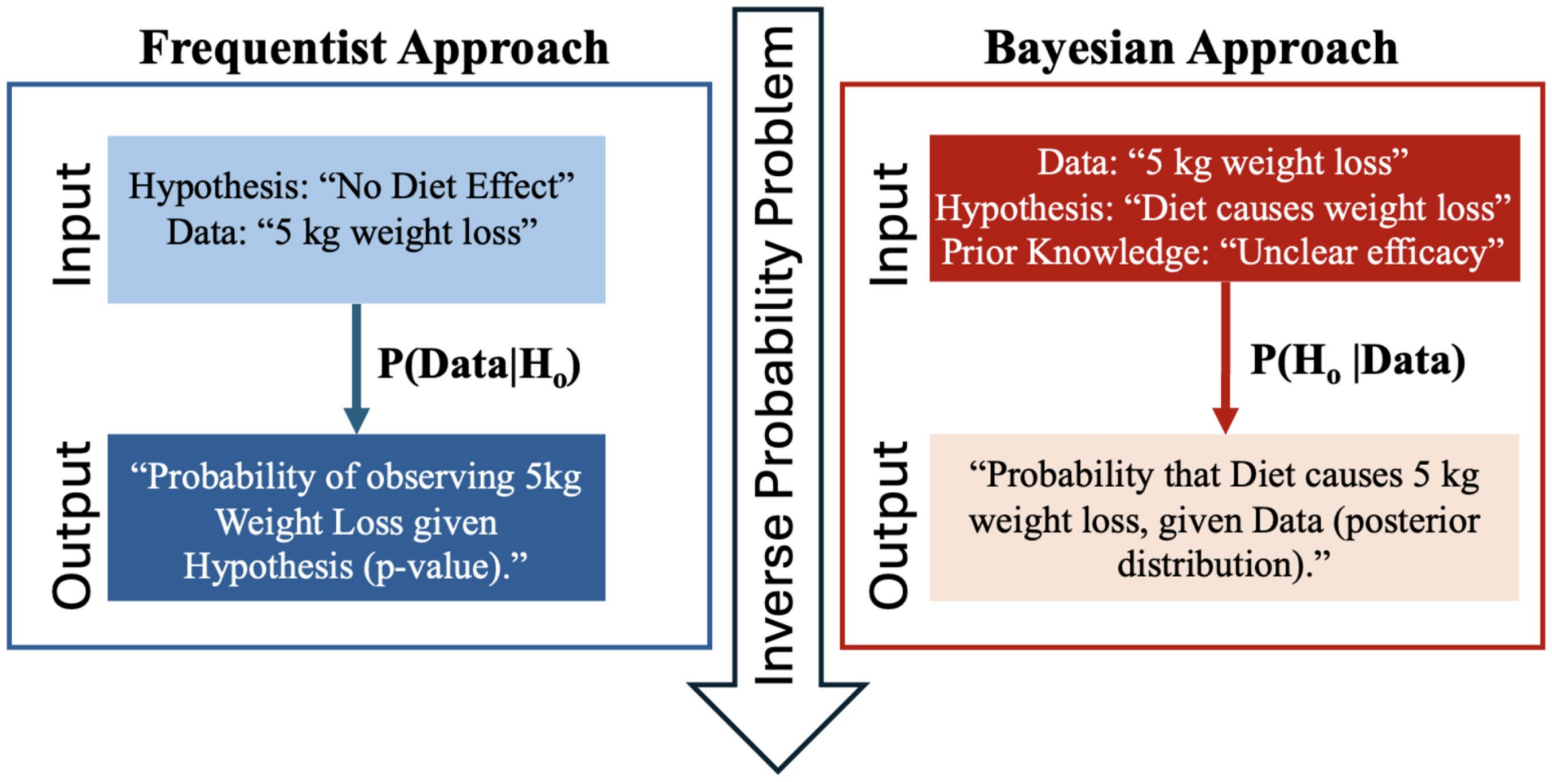
Illustration of the inverse probability problem: frequentist methods calculate P(data∣hypothesis ), whereas researchers often need P(hypothesis∣data) , which Bayesian methods provide by incorporating prior knowledge.

Frequentist inference treats model parameters as fixed and unknown, deriving conclusions solely from the sampling distribution of the data. Hypothesis tests are interpreted in terms of *p*-values and confidence intervals, which are measures that quantify the likelihood of extreme data under the null model but do not convey the probability that an effect exists. Moreover, frequentist methods rely on arbitrary significance thresholds (e.g., *p* < 0.05), which can mislead when interpreted categorically (8, 32–35). Bayesian inference instead treats parameters as random variables with probability distributions. Prior knowledge—whether informed by past studies, expert judgment, or biological plausibility—is formalized as a prior distribution, which is updated with data via Bayes’ theorem to produce a posterior distribution. This posterior captures our updated beliefs about the parameter’s likely values, enabling direct statements such as, “There is an 89% probability that the effect lies between −0.8 and −0.2.” Figure 1 represents this inverse probability problem on the example of weight loss due to diet. Specifically, it illustrates how frequentist methods compute the probability of the data given a hypothesis, whereas Bayesian inference directly addresses the probability of a hypothesis given the observed data, a distinction that profoundly impacts how evidence is interpreted.

To evaluate the robustness of inference across analytical paradigms, we employed both frequentist and Bayesian frameworks for longitudinal mixed-effects modeling. In the frequentist approach, models were fit using restricted maximum likelihood estimation, including fixed effects for dietary exposures, covariates, and sex with random intercepts for participants and study center. Two-sided hypothesis tests were conducted at an alpha level of 0.05, with 95% confidence intervals used to assess the precision of estimates.

Bayesian models were specified with a hierarchical structure to account for individual- and group-level variation. Parameter priors were weakly informative, typically drawn from normal or exponential distributions centered around plausible population values as outlined in prior work (26). Posterior distributions were estimated via Markov Chain Monte Carlo (MCMC) sampling using the ulam function from the *rethinking* package (12), which interfaces with the Stan probabilistic programming language (20, 24, 25, 36, 37). Models were run for 4,000 iterations across six chains. Convergence diagnostics, including effective sample size and the Gelman–Rubin R-hat statistic, were used to assess model stability. Inference was based on posterior means and 89% highest posterior density intervals, permitting probabilistic interpretation of parameter estimates. We provide formal model specifications for both approaches in the Supplementary Material, including extensions for hierarchical data structures.

### Covariate selection and causal assumptions

In congruence with previous work, we formalized causal assumptions, underpinning variable selection and model structure, using directed acyclic graphs (DAGs) constructed with the DAGitty software (38). These graphical models helped identify appropriate covariate adjustment sets while avoiding over adjustment or collider bias (15, 33–35). DAGs were constructed for each outcome to determine the minimally sufficient adjustment sets necessary to estimate the total effect of each dietary exposure and can be found in the Supplementary material.

### Baseline-adjusted models for longitudinal change

To specifically evaluate *change* in adiposity outcomes over time, we implemented baseline-adjusted longitudinal models. These models include the baseline value of each outcome (e.g., baseline BMI) as a covariate, enabling estimation of dietary associations with within-person change between baseline and follow-up. This approach isolates time-lagged effects by conditioning on initial status and is commonly used in longitudinal nutritional epidemiology to distinguish effects on change from cross-sectional associations (39–41). To explore these effects robustly, we again used both frequentist and Bayesian implementations. Frequentist and Bayesian baseline-adjusted models used the same model specification as the primary analysis, with the addition of baseline outcome as a covariate. Frequentist models were specified as linear mixed-effects models with fixed effects for dietary variables, covariates (including baseline outcome), and sex as well as random intercepts for individuals and study center. In the Bayesian framework, baseline-adjusted models were fit using the same hierarchical structure and priors as the primary models. Posterior distributions were interpreted in terms of the probability of a directional effect on outcome change and contrasted with unadjusted models to clarify the implications of baseline control. All models used standardized exposures to facilitate comparability, and full specification details are provided in the Supplementary material.

### Software and implementation

All analyses were conducted using R and Python. Data preprocessing and variable transformation were performed using base R and the *dplyr* package. Frequentist mixed-effects models were estimated using the *lme4* package. Bayesian models were implemented using the *rethinking*, and *rstan* packages. Time-lagged datasets were generated by forward-shifting outcome variables within each participant. Missing data were handled via complete case analysis. The datasets analyzed during the current study are available from the corresponding author upon reasonable request.

## Results

### Participant characteristics

**Figure 2:**
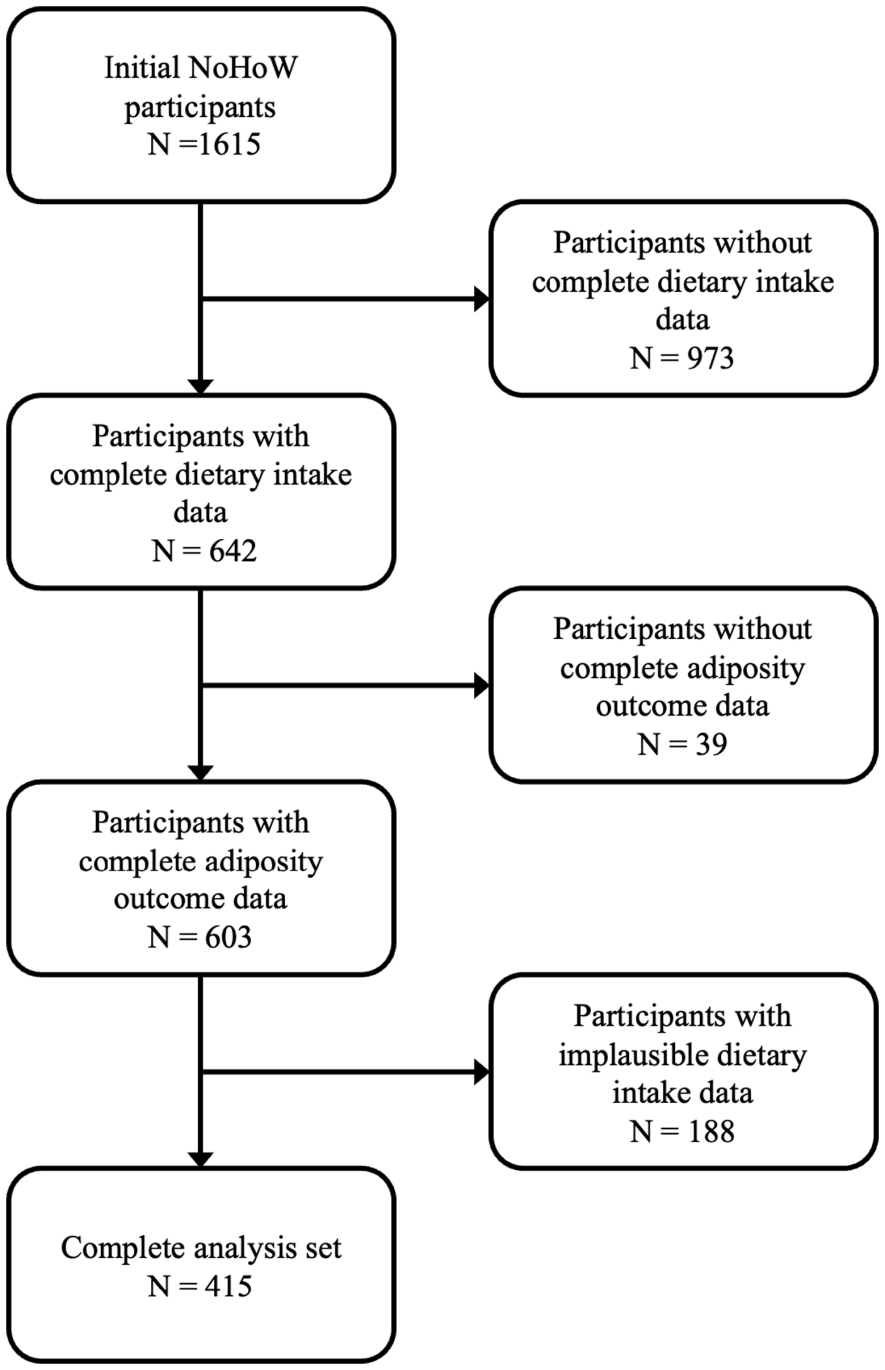
Flow diagram depicting participant inclusion and exclusion criteria for the final analysis set. Of the 1,615 initial NoHoW participants, 642 had complete dietary intake data at all time points. Of this subset, 603 had complete adiposity outcome data at all time points. Following exclusion of individuals with implausible dietary intake (*n* = 188) or incomplete outcome data (*n* = 39), a final sample of 415 participants was retained for analysis.

Baseline characteristics of the 415 participants included in the analysis are presented in Table 2. Figure 2 provides information detailing exclusion criteria and participant counts. Dietary intake values for non-milk extrinsic sugars (NMES), intrinsic sugars, starch, and fiber were stable across time points. The average NMES intake ranged from 21.8 to 24.8 grams per day across study centers, with intrinsic sugars averaging between 22.8 and 26.9 grams per day. Descriptive statistics for each carbohydrate type can be found in Table 2.

**Table 2:** Baseline characteristics of NoHoW (Navigating to a Healthy Weight) study participants.

Characteristic	*n*	Baseline	6 months	12 months	18 months
Age (years)	415	48.1 ± 11.7	48.6 ± 11.7	49.0 ± 11.7	49.5 ± 11.7
Sex (% male)	415	18.6	18.6	18.6	18.6
Dietary factors					
Energy (kcal)	415	1781 ± 594	1772 ± 530	1,656 ± 478	1,605 ± 472
Fat (g)	415	70.0 ± 32.1	69.7 ± 32.5	65.5 ± 29.6	63.0 ± 26.8
Saturated fat (g)	415	18.9 ± 11.3	18.9 ± 10.4	17.9 ± 10.6	17.1 ± 9.6
Protein (g)	415	89.6 ± 34.1	84.3 ± 27.8	80.2 ± 25.5	78.3 ± 25.5
Carbohydrate (g)	415	183.6 ± 72.3	188.6 ± 69.3	173.8 ± 64.6	169.4 ± 65.9
Fiber (g)	415	8.8 ± 6.9	8.8 ± 6.8	8.1 ± 6.2	7.9 ± 6.1
Intrinsic sugars (g)	415	26.9 ± 28.1	26.6 ± 26.9	23.7 ± 23.9	22.8 ± 22.7
NMES (g)	415	22.7 ± 21.7	24.8 ± 25.7	22.5 ± 23.4	21.8 ± 23.6
Starch (g)	415	68.7 ± 46.3	78.6 ± 53.7	71.2 ± 50.1	71.2 ± 51.7
Total sugars (g)	415	51.5 ± 42.2	53.1 ± 42.6	47.9 ± 39.9	45.7 ± 38.4
Alcohol (g)	415	8.6 ± 14.6	8.1 ± 14.4	7.6 ± 12.4	7.0 ± 12.8
Cholesterol (mg)	415	294 ± 193	266 ± 169	255 ± 152	253 ± 152
Anthropometric outcomes					
Body fat (%)	415	38.9 ± 8.3	37.8 ± 8.8	38.1 ± 8.4	38.6 ± 8.5
Waist circumference (cm)	415	92.5 ± 13.4	92.0 ± 12.7	92.2 ± 12.9	92.8 ± 13.1
BMI	415	29.1 ± 4.9	28.8 ± 4.7	29.1 ± 4.9	29.5 ± 5.0

Data are baseline means ± standard deviations. BMI, body mass index.

### Overview of model results

**Figure 3:**
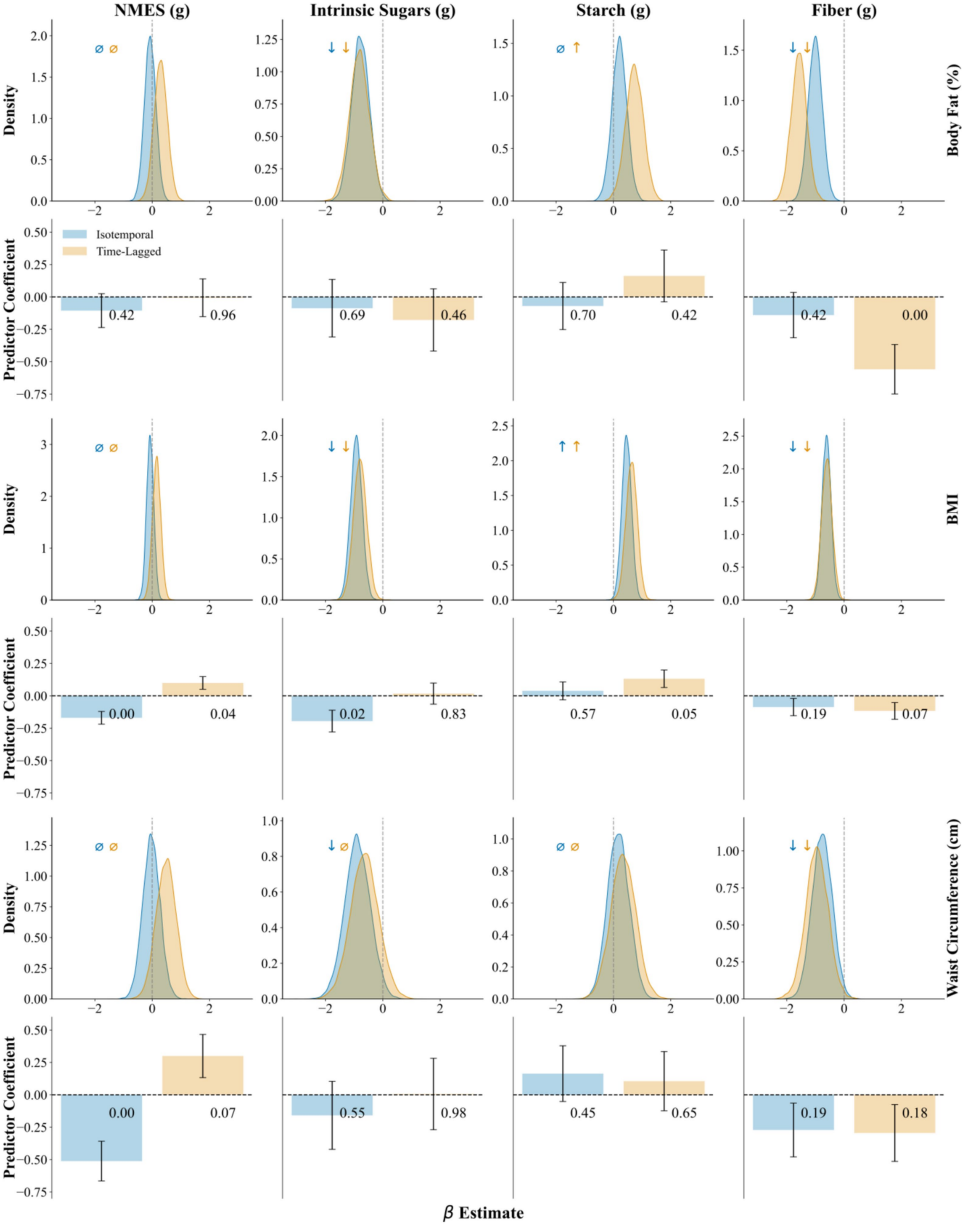
Non-baseline adjusted model Bayesian and frequentist associations of NMES, intrinsic sugars, starch and fiber with adiposity outcomes. Top row: Bayesian posterior distributions of effect size with 89% highest posterior density intervals (HPDI, shaded regions), arrows indicate directional credibility: ↓ = negative association (89% HPDI excludes zero), ↑ = positive association (89% HPDI excludes zero), *⌀* = non-credible (89% HPDI includes zero), bottom row: frequentist predictor coefficient with *p*-values on bar graphs with 95% confidence intervals (error bars) blue shaded region represents isotemporal models. Yellow Shaded Region represents time-lagged models.

**Figure 4:**
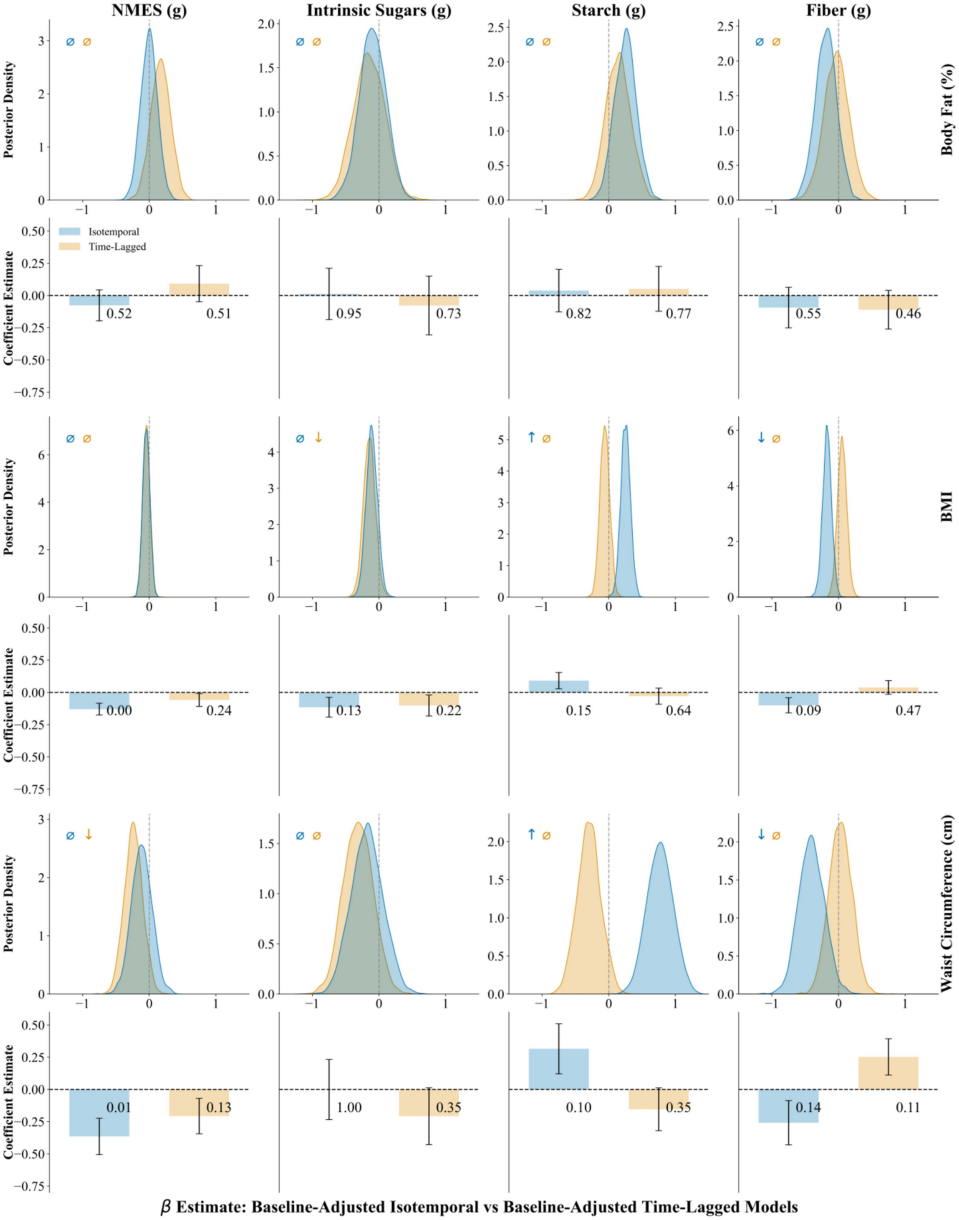
Baseline-adjusted models for longitudinal change. Time-lagged and isotemporal Bayesian and frequentist associations of NMES, intrinsic sugars, starch and fiber with adiposity outcomes adjusted for baseline measurements. Top row: Bayesian posterior distributions of effect size with 89% highest posterior density intervals (HPDI, shaded regions), arrows indicate directional credibility: ↓ = negative association (89% HPDI excludes zero), ↑ = positive association (89% HPDI excludes zero), *⌀* = non-credible (89% HPDI includes zero), bottom row: frequentist predictor coefficient with *p*-values on bar graphs with 95% confidence intervals (error bars) blue shaded region represents isotemporal models. Yellow Shaded Region represents time-lagged models.

Associations between dietary carbohydrate intake and measures of adiposity were evaluated using both isotemporal and time-lagged approaches across frequentist and Bayesian models. To assess whether associations were influenced by pre-existing differences in adiposity, we reran all models with adjustment for baseline outcome values. Baseline adjustment was outcome-specific, with each model controlling for its corresponding baseline measure (e.g., BMI models for baseline BMI, body-fat-percentage models for baseline body fat percentage) to isolate diet-related changes from confounding by initial adiposity. Figures 3, 4 provide a visual overview of the baseline-adjusted and unadjusted model results, including full posterior distributions and frequentist bar charts with error bars.

### Frequentist modeling

Table 3 presents the associations between intake of NMES, intrinsic sugars, starch, and fiber with indices of adiposity using frequentist linear mixed-effects models, both baseline-adjusted and unadjusted for baseline outcomes. Significant inverse associations (*p* < 0.05) were observed between NMES and both BMI and waist circumference in isotemporal models without baseline outcome adjustment, while intrinsic sugars also showed a significant inverse association with BMI. After adjusting for baseline adiposity, the intrinsic sugar–BMI association lost significance, whereas inverse NMES–adiposity associations persisted. In time-lagged models unadjusted for baseline outcomes, fiber intake was significantly inversely associated with body fat percentage, while NMES was positively associated with BMI. No significant associations were observed in baseline-adjusted time-lagged models.

**Table 3:** Associations of NMES, intrinsic sugars, starch and fiber intakes with indices of body fat (*n* = 415) from frequentist analysis.

		NMES			Intrinsic sugars			Starch			Fiber		
		Estimate	SE	*p*-value	Estimate	SE	*p*-value	Estimate	SE	*p*-value	Estimate	SE	*p*-value
Non-baseline-adjusted	Body fat (%)												
	Isotemporal	−0.11	0.13	0.42	−0.09	0.22	0.69	−0.07	0.18	0.70	−0.14	0.17	0.42
	Time-lagged	−0.01	0.15	0.96	−0.18	0.24	0.46	0.16	0.20	0.42	−0.56*	0.19	0.00
	BMI												
	Isotemporal	−0.17*	0.05	0.00	−0.19*	0.08	0.02	0.04	0.07	0.57	−0.09	0.07	0.19
	Time-lagged	0.10*	0.05	0.04	0.02	0.08	0.83	0.13	0.07	0.05	−0.12	0.06	0.07
	Waist circumference (cm)												
	Isotemporal	−0.51*	0.15	0.00	−0.16	0.26	0.55	0.16	0.22	0.45	−0.27	0.21	0.19
	Time-lagged	0.30	0.17	0.07	0.01	0.28	0.98	0.11	0.23	0.65	−0.29	0.22	0.18
Baseline-adjusted	Body fat (%)												
	Isotemporal	−0.08	0.12	0.52	0.01	0.20	0.95	0.04	0.16	0.82	−0.09	0.16	0.55
	Time-lagged	0.09	0.14	0.51	−0.08	0.23	0.73	0.05	0.17	0.77	−0.11	0.15	0.46
	BMI												
	Isotemporal	−0.13*	**0.05**	**0.00**	−0.12	0.08	0.13	0.09	0.06	0.15	−0.10	0.06	0.09
	Time-lagged	−0.06	0.05	0.24	−0.10	0.08	0.22	−0.03	0.06	0.64	0.04	0.05	0.47
	Waist circumference (cm)												
	Isotemporal	−0.36*	**0.14**	**0.01**	0.00	0.23	1.00	0.32	0.19	0.10	−0.26	0.17	0.14
	Time-lagged	−0.21	0.14	0.13	−0.21	0.22	0.35	−0.15	0.17	0.35	0.25	0.14	0.11

^*^Indicates significant results ( α=0.05).

Results of linear mixed effects regression (lmer) model. Estimates, standard error, and *p*-value are given for each combination of predictor and outcome. Each model adjusted for age, sex, protein, alcohol, saturated fat, fiber, and remaining energy. Participant effect and intervention center are used as random effects in the lmer model. Most significant values in bold.

### Bayesian modeling

Table 4 reports baseline-adjusted and unadjusted Bayesian model associations between NMES, intrinsic sugars, starch, and fiber intakes and the three adiposity measures. In models without baseline outcome adjustment (both isotemporal and time-lagged), higher intrinsic sugar and fiber intakes were inversely associated with body fat percentage, BMI, and waist circumference. Starch showed positive associations with BMI and body fat percentage, primarily in time-lagged models, while NMES was unrelated to any outcome. The direction and magnitude of associations for starch and fiber effects increased in time-lagged models.

**Table 4:** Associations of NMES, intrinsic sugars, starch and fiber intakes with indices of body fat (*n* = 415) from Bayesian analysis.

		NMES		Intrinsic sugars		Starch		Fiber	
		Mean	*⌀*, ↑, ↓	Mean	*⌀*, ↑, ↓	Mean	*⌀*, ↑, ↓	Mean	*⌀*, ↑, ↓
Non-baseline-adjusted	Body fat (%)								
	Isotemporal	−0.1	*⌀* (−0.4, 0.3)	**−0.8**	**↓ (−1.3, −0.3)**	0.2	*⌀* (−0.2, 0.6)	**−1.0**	**↓ (−1.4, −0.6)**
	Time-lagged	0.3	*⌀* (−0.1, 0.7)	**−0.8**	**↓ (−1.3, −0.2)**	**0.7**	**↑ (0.2, 1.2)**	**−1.6**	**↓ (−2.0, −1.2)**
	BMI								
	Isotemporal	−0.1	*⌀* (−0.3, 0.1)	**−0.9**	**↓ (−1.2, −0.6)**	**0.5**	**↑ (0.2, 0.7)**	**−0.6**	**↓ (−0.9, −0.4)**
	Time-lagged	0.2	*⌀* (−0.1, 0.4)	**−0.8**	**↓ (−1.2, −0.4)**	**0.6**	**↑ (0.3, 0.9)**	**−0.6**	**↓ (−0.9, −0.3)**
	Waist circumference (cm)								
	Isotemporal	−0.0	*⌀* (−0.5, 0.5)	**−0.9**	**↓ (−1.6, −0.2)**	0.2	*⌀* (−0.4, 0.8)	**−0.8**	**↓ (−1.3, −0.2)**
	Time-lagged	0.5	*⌀* (−0.1, 1.0)	−0.6	*⌀* (−1.6, 0.3)	0.3	*⌀* (−0.4, 1.0)	**−1.0**	**↓ (−1.6, −0.4)**
Baseline-adjusted	Body fat (%)								
	Isotemporal	0.00	*⌀* (−0.2, 0.2)	−0.09	*⌀* (−0.4, 0.2)	0.25	*⌀* (0.0, 0.5)	−0.19	*⌀* (−0.5, 0.1)
	Time-lagged	0.17	*⌀* (−0.1, 0.4)	−0.15	*⌀* (−0.5, 0.2)	0.14	*⌀* (−0.1, 0.4)	−0.03	*⌀* (−0.3, 0.3)
	BMI								
	Isotemporal	−0.04	*⌀* (−0.1, 0.0)	−0.11	*⌀* (−0.2, 0.0)	**0.25**	**↑ (0.1, 0.4)**	**−0.17**	**↓ (−0.3, −0.1)**
	Time-lagged	−0.04	*⌀* (−0.1, 0.0)	**−0.15**	**↓ (−0.3, 0.0)**	−0.06	*⌀* (−0.2, 0.1)	0.05	*⌀* (−0.1, 0.2)
	Waist circumference (cm)								
	Isotemporal	−0.11	*⌀* (−0.3, 0.1)	−0.18	*⌀* (−0.6, 0.2)	**0.77**	**↑ (0.5, 1.1)**	**−0.40**	**↓ (−0.7, −0.1)**
	Time-lagged	**−0.24**	**↓ (−0.5, 0.0)**	−0.31	*⌀* (−0.7, 0.0)	−0.30	*⌀* (−0.6, 0.0)	0.03	*⌀* (−0.2, 0.3)

Values are means (89% CIs) of β estimate posterior distributions. Transformations of variables for analysis: *z*-scores of logs of intakes in kcal. The model adjusted for age, sex, participant effect, intervention center, protein, alcohol, saturated fat, fiber, and remaining energy. Arrows (↑, ↓) indicate significant associations (89% HPDI did not include zero), i.e., significant increases or decreases in the outcome observed in 89% of the samples. *⌀* indicates non-significant findings (HPDI intervals included zero) of NMES, intrinsic sugars, starch and fiber intakes with indices of body fat (*n* = 415). Most significant values in bold.

In baseline-adjusted isotemporal models, starch was positively associated with BMI and waist circumference, whereas fiber was inversely associated with both measures. In baseline-adjusted time-lagged models, intrinsic sugar was inversely associated with BMI, and NMES with waist circumference.

### Baseline adjustment

Effect estimates were substantially attenuated after adjustment for baseline adiposity. Strong associations observed in the models without adjustment for baseline outcomes, particularly for fiber and starch predictors and body fat outcomes, were no longer statistically significant and demonstrated reduced evidence in both Bayesian and frequentist frameworks. The direction of associations was generally preserved in relationships between intrinsic sugars and adiposity measures, but effect sizes shrank considerably, and statistical significance was lost in all but one case. For fiber and starch, the adjustment led to near-complete attenuation of effects, barring a single new protective association.

## Discussion

This study systematically compared the effects of carbohydrate subtype intake on adiposity using both isotemporal and time-lagged modeling frameworks under frequentist and Bayesian inference. A key contribution of this work lies in its cross-framework comparison of modeling strategies, temporal and inferential, which has rarely been done in nutritional epidemiology despite longstanding concerns over causal misinterpretation in observational studies (3, 6, 8).

### Temporal frameworks: isotemporal and time-lagged

Directional agreement was observed across models without baseline adjustment in both temporal frameworks for all associations but that between NMES and BMI, with discrepancies limited to shifts between statistical significance and non-significance. However, notable shifts in signal clarity and magnitude between carbohydrate subtypes emerged with change to temporal framework for the models unadjusted for baseline outcomes. Time-lagged models more consistently identified statistically significant associations and amplified the magnitude of protective or adverse effects concerning starch and fiber predictors. This was most evident in associations with body fat: here, the time-lagged Bayesian model identified a significant adverse association with starch intake that was not detected in isotemporal models. Similarly, the protective association between fiber intake and body fat was strengthened under the time-lagged Bayesian framework. Although most prominent in body fat outcomes, similar but attenuated effects were observed for starch and fiber associations with all anthropometric outcomes, suggesting that these components may exert measurable effects over time that are muted or obscured in concurrent analyses. Time-lagged frequentist models similarly identified significant associations for starch and fiber, reinforcing these findings.

Conversely, some inverse associations observed with isotemporal models without baseline adjustment lost statistical support when temporal precedence was enforced, as seen in the association between intrinsic sugars and BMI. These changes were observed in both Bayesian and frequentist modeling frameworks for intrinsic and extrinsic sugar relationships, suggesting that they reflect structural shifts in the modeling paradigm rather than methodological artifacts.

In baseline-adjusted models, temporal specification produced a divergence for starch and fiber predictors. Isotemporal models identified significant associations of both predictors with BMI and waist circumference, whereas time-lagged models detected none. Indeed, the time-lagged specification even suggested a weak protective trend between starch and waist circumference—opposite in direction to the isotemporal result, though still nonsignificant. These discrepancies highlight the sensitivity of inference to temporal modeling choices and raise the possibility of phenomena akin to Lord’s paradox, in which adjustment for baseline measures alters or reverses observed associations.

Explicitly modeling temporal dynamics offers alternative statistical insights. The clearer signals detected in non-baseline-adjusted models with time-lagged data for starch and fiber intake predictors suggest that temporally structured analyses may more faithfully capture the directionality of certain diet–health relationships, consistent with recent longitudinal modeling approaches in physical activity and nutrition research (8, 9, 34). For instance, Hliang-Hliang et al. (42) found temporal associations between diet and some non-communicable diseases that were more prominent in lagged generalized estimation equations analyses relative to time-invariant or standard time-varying approaches. However, the absence of similarly enhanced signals for intrinsic and extrinsic sugars in time-lagged non-baseline-adjusted models and the divergence of starch- and fiber-adiposity associations in baseline-adjusted models underscores the importance of aligning modeling approaches with the expected temporal dynamics of specific diet-health mechanisms.

Our findings reinforce the critique that isotemporal models may obscure or invert exposure–outcome relationships due to reverse causality and residual confounding (7, 9), which can arise when early manifestations of subclinical disease influence reported dietary behaviors, but these changes may be most visible in models not adjusted by baseline outcome. By introducing a temporal buffer between dietary assessment and the start of follow-up for disease outcomes, time-lagged models help ensure that exposure temporally precedes outcome onset. While this does not establish causality, it reduces the likelihood that observed associations are driven by pre-existing but undiagnosed etiologies.

### Statistical models: frequentist and Bayesian approaches

Bayesian hierarchical models, as employed here, offer a promising alternative to traditional methods. The incorporation of prior knowledge, whether biological plausibility or meta-analytic evidence, and accommodation of complex study designs and correlated data (16) allow more robust treatment of low sample size, more reliable handling of noise in sample data, and improved estimation in the presence of non-normal distributions. Advances in probabilistic programming have made Bayesian approaches increasingly accessible and scalable (19), yet adoption in nutrition remains limited. This study demonstrates a practical implementation of a Bayesian approach, bridging the gap between theoretical advocacy and applied use in an epidemiological context (13).

The Bayesian models provided interpretable estimates and credible intervals with consistent sensitivity behavior. As shown in Figure 3, the posterior distributions for fiber and intrinsic sugars were generally unimodal, narrow, and centered well away from zero, supporting both the precision and stability of these estimates. Compared to frequentist models, Bayesian estimates were more stable across temporal frameworks, with fewer shifts in significance and no changes in direction among significant associations. However, regularization through weakly informative priors can improve model stability and reduce sensitivity to outliers but may also introduce shrinkage bias toward null effects particularly by weak to moderate associations (43). To test this, we conducted a sensitivity analysis by using a subset of the models and found near-identical posteriors for uniform and normal priors.

Bayesian models tended to yield more statistically significant results compared to frequentist methods, as was particularly evident for fiber and intrinsic sugars. Consistent with prior expectations, Bayesian NMES estimates show evidence of shrinkage toward the null, suggesting that the models regularized potentially unstable associations seen in frequentist analysis. This aligns with previous work showing inconsistent relationships between NMES and adiposity (26, 44, 45), and may indicate noise sensitivity in observational dietary data.

### Adjusting for baseline adiposity

To verify that our time-lagged signals were not simply artifacts of unadjusted baseline differences, we reran all models with corresponding baseline outcome values (i.e., baseline BMI for BMI models) included as a covariate (Figure 4). Adjustment for baseline adiposity led to marked attenuation of the fiber– and starch–body fat associations, which lost statistical support in both Bayesian and frequentist frameworks. Considered with the near-complete loss of significance for fiber and intrinsic sugar associations, it is demonstrated that unadjusted models can overstate dietary impacts when pre-existing adiposity is unevenly distributed. Similar attenuation has been reported in other large prospective cohort studies when transitioning from unadjusted to baseline-adjusted models, particularly for exposures such as dietary fiber and sugars (46, 47). Conversely, baseline adjustment revealed modest associations of NMES with waist circumference and starch with BMI, indicating potential effects that were obscured in unadjusted models. Including baseline adiposity is therefore essential to isolate true diet-change effects from confounding by initial weight.

### Implications of modeling strategy in nutritional epidemiology

Our results yield substantive methodological implications for applied nutrition research. Temporal misalignment and inferential limitations have been repeatedly identified as threats to validity in nutritional epidemiology (3). By directly comparing isotemporal and time-lagged models within both Bayesian and frequentist paradigms, we find that analytical framework meaningfully alters effect size, direction, and interpretability. This confirms earlier simulation and applied work showing that the interaction between modeling strategy and data structure can substantially shape inferences, especially in the context of longitudinal, high-dimensional data (36).

Time-lagged models are specifically suited to examining temporality and the directionality of associations, as they ensure that exposure precedes outcome and enable the investigation of delayed dietary effects. However, while such temporal separation strengthens the plausibility of hypothesized causal pathways, it is essential to acknowledge that these models do not establish causality in the absence of randomized experimental validation or auxiliary methodological approaches. Thus, observed associations should be interpreted as suggestive but not definitive evidence for causal effects. Isotemporal models, as employed in this study, estimate exposure–outcome associations using concurrent measurements, but do not address substitution effects or provide direct estimates for dietary reallocation. Researchers specifically interested in quantifying the impact of substituting one dietary component for another should employ isotemporal substitution models, which are purpose-built for that objective and yield estimates directly interpretable for clinical and public health guidance.

In practice, we recommend that the choice of modeling framework be guided by specific study objectives. Isotemporal models are optimal for assessing concurrent associations, while time-lagged models should be utilized when temporal ordering or delayed exposures are of primary interest. Time-lagged models may be valuable in informing hypothesis generation regarding causal pathways. Importantly, we show that time-lagged models not adjusted for baseline outcome values can overstate dietary impacts when pre-existing adiposity is unevenly distributed. Including baseline adiposity is therefore essential to isolate true diet change effects from confounding by initial weight. Where data structure allows, implementing both approaches in parallel may provide complementary perspectives and enhance confidence in the robustness of findings. This comparative approach is especially relevant in high-dimensional nutritional data, where the risk of misinterpretation due to model misspecification remains significant and the implications for public health guidance are substantial.

Several limitations should be acknowledged. First, the sample size of the NoHoW dataset is relatively small leading to potentially increases of margin of error and risk of type I or type II errors. Second, self-reported dietary intake is inherently prone to misreporting, recall bias, and social desirability bias, which can attenuate true associations or introduce differential misclassification. Although energy adjustment and covariate control help mitigate some of these biases, they do not eliminate concerns about the validity of self-reported data. Third, variation in food processing and matrix effects may modulate associations beyond nutrient subtype alone. Additionally, limiting the analysis to participants with complete data could introduce selection bias if missingness is related to exposures or outcomes. This potential bias should be considered when interpreting the study’s generalizability and potential causal links. We acknowledge that the large number of comparisons increases the potential for type I error. However, given that our primary objective was methodological comparison rather than establishing definitive diet-health associations, and that identical statistical comparisons were applied uniformly across all four modeling frameworks, any inflated type I error rate would not differentially affect the cross-method comparisons central to our findings. Finally, although the 6-month lag interval may have helped identify temporally plausible associations, it may still overlook shorter-term metabolic dynamics or seasonal dietary variations. Future work incorporating finer temporal resolution, or repeated biomarker data (e.g., CGM) could yield more granular insights into the time course of dietary effects.

## Conclusion

This study demonstrates that both temporal structure and statistical framework substantially influence observed associations between dietary intake and health outcomes in nutritional epidemiology. By comparing isotemporal and time-lagged approaches in both frequentist and Bayesian contexts, hierarchical Bayesian models offer more stable estimates and robust evidence for associations relative to the frequentist methods evaluated alone. For causal inference, time-lagged models can provide additional insight to the directionality of the predictors and outcomes, but this cannot be verified without additional experimental validation. Moreover, models unadjusted for baseline adiposity can overstate dietary impacts when pre-existing adiposity is unevenly distributed; including baseline adiposity measures is therefore essential to isolate true diet-change effects from confounding by initial weight.

Regardless of the statistical paradigm, the analytical strategy, including the choice of temporal framework (isotemporal versus time-lagged), should be pre-specified based on the underlying etiological hypotheses. If frequentist methods are employed, comparing results from isotemporal and time-lagged analyses can serve as a valuable sensitivity analysis to assess the consistency of effects across different temporal assumptions. Bayesian analysis appears preferable for complex analysis of FFQ data as obtained results are more consistent across temporal frameworks.

## Data Availability

The original contributions presented in the study are included in the article/Supplementary material, further inquiries can be directed to the corresponding author.
