# Supplementary material for: Modeling frameworks in nutritional epidemiology matter: comparing isotemporal and time-lagged Bayesian and frequentist approaches of carbohydrate intake and adiposity
Source: Front Nutr. 2025 Dec 18;12:1700898. doi: 10.3389/fnut.2025.1700898 (PMC12756090; doi:10.3389/fnut.2025.1700898)
Supplement: Supplementary file 1 [file Supplementary_file_1.docx]

*Supplementary Information*

**Comparing Isotemporal and Time-Lagged Association Analyses: A Cross-Study Evaluation Using Frequentist and Bayesian Methods**

Table of Contents

[SI Figure 1 Directed Acyclic Graphs 2](#_Toc206411818)

[Bayesian Model Specification 3](#_Toc206411819)

[Frequentist Hierarchical Model Specification 5](#_Toc206411820)

# SI Figure 1 Directed Acyclic Graphs

DAGs visualize the complex causal relationships between nutritional variables and health outcomes. This DAG was developed through a systematic process, incorporating current scientific knowledge and expert consensus in nutritional science. Each node represents a variable of interest, such as anthropometric measures, while the directed edges indicate hypothesized causal paths based on existing literature. Confounders with potential backdoor paths are carefully identified and depicted in orange to highlight their role in the analysis. This graphical representation facilitates the application of Bayesian inference by explicitly modeling the dependencies and independencies among variables. The transparency of DAGs allows for clear communication of the assumptions underlying our causal models, enabling replication and validation in future studies. Additionally, the careful distinction between predictors, outcomes, and confounders aids in the accurate interpretation of the results, ensuring robust and credible conclusions in our investigation of nutritional impacts on health. The DAG for the NoHoW model used in this paper is shown below in Figure S1.

Figure S1 Directed Acyclic Graphs (DAG) of NoHoW model. Predictors in green on left. Each predictor may be used for each outcome, but remaining energy variable is updated accordingly. Confounders with backdoor paths are in orange. Outcomes in blue on the right. Arrows indicate flow of information, with black arrow indicating the causal impact of predictor on outcome of interest. Light green and blue shaded boxes indicate alternative predictors and outcomes.

# Bayesian Model Specification

The Bayesian model specification for the NoHoW model, detailed below, outlines the statistical framework employed in our analysis. This model specification is provided to ensure transparency and reproducibility of our results. By clearly defining the likelihood, linear predictor, and priors, we offer a comprehensive view of the assumptions and structure underlying our inferential approach. The inclusion of this detailed specification allows other researchers to understand the exact methods used, facilitates replication of the study, and enables critical evaluation of the model’s robustness. The normal priors chosen for the coefficients reflect a non-informative stance, assuming no prior knowledge about the direction or magnitude of the effects, which is essential for unbiased parameter estimation. Moreover, the use of standardized variables ensures comparability across different predictors, simplifying the interpretation of the model coefficients. Providing this detailed specification aligns with best practices in Bayesian statistics, promoting rigorous scientific inquiry and enhancing the credibility of our findings.

Let $Y_{i}$ denote the outcome of interest. The model can be specified as follows:

*Likelihood*

$Y_{i} \sim N(\mu_{i},\sigma)$

where $\mu_{i}$ is the mean and σ denotes the residual standard deviation of the normal distribution for the i-th observation.

*Linear Predictors*

$$\mu_{i}=\alpha_{sex[s]}+\alpha_{center\_id[j]}+\alpha_{Participant\left[ k \right]}+\beta_{predictor}*predictor_{i} +\beta_{protein}*protein+\beta_{alcohol}+alcohol+ \beta_{fat}*fat+ \beta_{age}*age_{i}+\beta_{remaining\_energy}*remaining\_energy$$

$$\alpha_{Participant\left[ k \right]}=\mu_{Participant}+ \sigma_{Participant}*a_{raw\_participant[k]}$$

*Priors*

The priors for the parameters are specified as follows:

$$\alpha_{sex[s]} \sim N(0,1)$$

$$\alpha_{raw\_participant[k]} \sim N(0,1)$$

$$\beta_{predictor} \sim N(0,1)$$

$$\beta_{protein} \sim N(0,1)$$

$$\beta_{alcohol} \sim N(0,1)$$

$$\beta_{fat} \sim N(0,1)$$

$$\beta_{remaining\_energy} \sim N(0,1)$$

$$\mu_{participant} \sim N(0,1)$$

$$\sigma\sim Exponential(1)$$

$$\sigma_{Participant} \sim Exponential(1)$$

*Notation*

- $Y_{i}$: Outcome for the i-th observation
- $\mu_{i}$: Mean of the normal distribution for the i-th observation
- $\sigma$: Standard deviation of the normal distribution (assumed to be constant across observations)
- $\mu_{Participant}$: Mean of the participant-level effect
- $\sigma_{Participant}$: Standard deviation of the participant effect
- $\alpha_{sex\left[ s \right]}$: Fixed effects intercept term for each sex g (male, female)
- $\alpha_{center\_id\left[ j \right]}$: Fixed effects intercept term for the j-th center
- $\alpha_{raw\_participant[k]}$: Intercept term for the k-th participant (random effect)
- $\alpha_{Participant\left[ k \right]}$: Participant-specific intercept (partially pooled)
- $\beta_{predictor}$: Coefficient for the predictor variable
- $\beta_{protein}$: Coefficient for the protein variable
- $\beta_{alcohol}$: Coefficient for the alcohol variable
- $\beta_{fat}$: Coefficient for the saturated fat variable
- $\beta_{age}$: Coefficient for the age variable
- $\beta_{remaining\_energy}$: Coefficient for the energy variable

Since all the predictors and outcomes were standardized, normal priors with a mean of 0 were employed for the intercept ($\alpha$) and the coefficients ($\beta$). The choice of a mean of 0 for these priors reflects the assumption that, in the absence of data, the predictor variables do not have a systematic effect on the outcome.

This model structure incorporates repeated measures through a hierarchical approach, where participant-specific intercepts $\alpha_{Participant[k]}$ are treated as random effects and modeled using a non-centered parameterization. $\mu_{Participant}$ represents the overall mean effect pooled across participants, while $\sigma_{Participant}$ captures the variability between participants. This pooling of information allows the model to borrow strength across individuals, improving the stability and precision of the estimates. The non-centered parameterization is facilitated by the introduction of the auxiliary variable $\alpha_{raw\_participant[k]}$, which follows a standard normal distribution. This approach enhances the model’s flexibility and computational efficiency, particularly in Bayesian inference, by improving the convergence properties of the sampling algorithm. By including $\alpha_{Participant[k]}$ as a random effect, the model effectively controls for clustering within the same participant, accounting for the repeated measurements and individual-level variability. This results in more robust and reliable estimates of the effects of interest.

# Frequentist Hierarchical Model Specification

This document outlines the general frequentist hierarchical model specification corresponding to the series of linear mixed-effects models fitted using the lmer function from the lmerTest package in R. The purpose is to provide a clear and reproducible statistical framework for the analysis from a frequentist perspective.

The models described in the R code share a common structure, differing only in the primary carbohydrate predictor and the specific adiposity outcome. The following specification is a generalized representation of all fitted models.

**Model Formulation**

Let $Y_{i}$ be the outcome variable for observation $i$. The model is specified as follows:

**Distributional Assumption**

The outcome variable is assumed to be normally distributed, conditional on the fixed and random effects. The errors, $\varepsilon_{i}$, are assumed to be independent and identically distributed following a normal distribution with a mean of 0 and a constant variance $\sigma_{\varepsilon}^{2}$ (to $\sigma_{\varepsilon}^{2}$ is equivalent to σ² in the Bayesian model),

$Y_{i}=\mu_{i}+\varepsilon_{i}$ where $\varepsilon_{i}\sim N\left( 0,\sigma_{\varepsilon}^{2} \right)$

**Linear Predictor**

The expected value of the outcome, $\mu_{i}$, is modeled as a linear combination of fixed effects (for the population) and random effects (for specific groups).

$$\mu_{i}=\alpha+\beta_{predictor}\cdot Predictor_{i}+\beta_{energy} \cdot Energy_{i} + \beta_{protein} \cdot Protein_{i}+\beta_{fiber}\cdot Fiber_{i}+\beta_{fat}\cdot Fat_{i}+\beta_{age} \cdot Age_{i}+\beta_{sex} \cdot{Sex}_{i}+ \mu_{c\left[ i \right]} + v_{p\left[ i \right]}$$

Note: In models where a specific nutrient (e.g., fiber) is the primary predictor of interest, its corresponding term is omitted from the list of general covariates to avoid collinearity.

**Random Effects Structure**

The random effects are assumed to be random variables that follow a normal distribution with a mean of 0 and a variance that is estimated from the data. They are assumed to be independent of the residual errors.

$v_{p}\sim N(0,\sigma_{participant}^{2})$ for each participant $p$

**Parameter Estimation**

In the frequentist framework, there are no prior distributions for the parameters. Instead, the fixed-effects parameters ($\alpha$, $\beta_{k}$​) and the variance components ($\sigma_{\varepsilon}^{2}$​, $\sigma_{participant}^{2}$​) are treated as fixed, unknown constants. These parameters are estimated from the data, typically using Maximum Likelihood (ML), as specified by REML = FALSE. Which allows for comparison between models with different fixed effects. The goal is to find the parameter values that maximize the likelihood of observing the actual data. Inference is then based on these point estimates, their standard errors, and confidence intervals.

*Notation*

- $Y_{i}$: Outcome for the i-th observation
- $\mu_{i}$: Mean of the normal distribution for the i-th observation

- $\varepsilon_{i}$: The residual error for the i-th observation

- $\sigma_{\varepsilon}^{2}$: The variance of the residual errors
- $\alpha,\beta_{k}$: Fixed-effects parameters (intercept and slopes) to be estimated
- $Predictor_{i}$: The primary standardized predictor
- $c_{\left[ i \right]}$: The center_id (included as a categorical fixed effect) corresponding to the i-th observation
- $p_{\left[ i \right]}$: The participant_id corresponding to the i-th observation
- $v_{p}$: The random intercept for participant p
- $v_{p[i]}$​: The random intercept associated with the participant at i-th observation
- $\sigma_{participant}^{2}$: The variance of the participant-level random intercepts

This hierarchical model structure accounts for the non-independence of observations by including random intercepts for participants. This allows the model to estimate distinct baseline values for each participant, effectively controlling for data clustering by partitioning the total variance into different sources.
